# Supplementary material for: Disclosure of conventional and complementary medicine use to medical doctors and complementary medicine practitioners: A survey of rates and reasons amongst those with chronic conditions
Source: PLoS One. 2021 Nov 4;16(11):e0258901. doi: 10.1371/journal.pone.0258901 (PMC8568289; doi:10.1371/journal.pone.0258901)
Supplement: S1 File — Complete questionnaire for the CAMUHLD survey 2017. (PDF) [file pone.0258901.s001.pdf]

Survey copy – complete questionnaire for project:

Complementary medicine use, health literacy and disclosure in the Australian population

---

Information about this research

WHO IS DOING THE RESEARCH?
My name is Dr Erica McIntyre and I am a research associate from the School of Psychology at Charles Sturt University. My colleagues are Dr Amie Steel, a postdoctoral research fellow from the Australian Research Centre in Complementary and Integrative Medicine (ARCCIM) at UTS, and Associate Director-Research at Endeavour College of Natural Health, and Dr Joanna Harnett, Associate Lecturer from the Faculty of Pharmacy at the University of Sydney.

WHAT IS THIS RESEARCH ABOUT?

This research is to find out about the patterns of complementary use in the Australian population. It is also designed to explore the understanding and communication of complementary medicine use in Australia.

IF I SAY YES, WHAT WILL IT INVOLVE?

We will ask you to complete an online questionnaire that may take up to 15 minutes to complete. Your completion of this survey will be taken as your consent to participate.

ARE THERE ANY RISKS/INCONVENIENCE?

Yes, there is some inconvenience. This survey will take up to 15 minutes to complete.

WHY HAVE I BEEN ASKED?

You are a member of the Australian general population.

DO I HAVE TO SAY YES?

. No. Your participation is completely voluntary.

WHAT WILL HAPPEN IF I SAY NO?

Nothing. We will not contact you about this research again.

IF I SAY YES, CAN I CHANGE MY MIND LATER?

You can change your mind at any time and you do not have to say why. We won't contact you about this research again.

WHAT IF I HAVE CONCERNS OR A COMPLAINT?

If you have concerns about the research that you think I or my colleagues can help you with, please feel free to contact me at emcintyre@csu.edu.au.

If you would like to talk to someone who is not connected with the research, you may contact the Research Ethics Officer on 02 9514 9772, and quote this number (20170242)

Consent to participate

I agree to participate in the research project Complementary medicine use, literacy and disclosure in the Australian population (#20170242) being conducted by Dr Erica McIntyre (emcintyre@csu.edu.au).

I understand that the purpose of this study is to explore the patterns of complementary medicine use in the Australian general population as well as the understanding and communication of complementary medicine use in Australia.

I understand that I have been asked to participate in this research because my behaviours and perspectives as a member of the Australian population is valuable, and that my participation in this research will involve completing an online survey lasting approximately 15 minutes.

I am aware that I can contact Dr Erica McIntyre if I have any concerns about the research. I also understand that I am free to withdraw my participation from this research project at any time I wish, without consequences, and without giving a reason.

I agree that I have had an opportunity to have all of my questions answered fully and clearly.

I agree that the research data gathered from this project may be published in a form that does not identify me in any way.

1) Do you agree with all of the above and consent to participate in this research project?

( ) Yes

( ) No

Section 1: Demographics

About You

2) What is your gender? Please select the response that best applies to you.

( ) Female

( ) Male

( ) Unspecified

3) What is your age range?

( ) 18-29

( ) 30-39

( ) 40-49

( ) 50-59

( ) 60 and over

4) What is your residential postcode?

____

5) How do you manage financially at the moment? (Mark one only)

( ) It is impossible

( ) It is difficult all of the time

( ) It is difficult some of the time

( ) It is not too bad

( ) It is easy

6) What is the highest qualification you have completed?

( ) No formal qualifications

( ) Year 10 or equivalent

( ) Year 12 or equivalent

( ) Trade/apprenticeship

( ) Certificate/diploma

( ) University degree

( ) Higher university degree (e.g. Masters, PhD)

7) Do you currently have private health insurance?

( ) Yes

( ) No

8) Do you currently have a Health Care Card?

( ) Yes

( ) No

9) What best describes your employment status? (Mark one only)

( ) Full time work (35 or more hours per week)

( ) Part time work (less than 35 hours per week)

( ) Casual/temp work (irregular hours)

( ) Looking for work

( ) Not in the paid workforce

10) What is your present marital status?

( ) Never married

( ) Married

( ) De facto (opposite sex)

( ) De facto (same sex)

( ) Separated

( ) Divorced

( ) Widowed

11) If you currently have private health insurance for ancillary services, please indicate which services are covered:

[ ] I do not have private health insurance for ancillary services

[ ] Yoga/pilates/mediatation

[ ] Physiotherapy

[ ] Psychology services

[ ] Chiropractic

[ ] Osteopathy

[ ] Acupuncture

[ ] Chinese medicine/herbs

[ ] Homeopathy

[ ] Naturopathy

[ ] Western herbal medicine

[ ] Remedial massage/massage therapy

[ ] Nutrition/dietetics

Section 2:

About your health and wellbeing

The following questions ask you how satisfied you feel, on a scale from zero to 10. Zero means you feel no satisfaction at all and 10 means you feel completely satisfied.

12) Thinking about your own life and personal circumstances, how satisfied are you with your life as whole?

0 _________________________[__]______________________________ 10

13) How satisfied are you with your standard of living?

0 _________________________[__]______________________________ 10

14) How satisfied are you with your health?

0 _________________________[__]______________________________ 10

15) How satisfied are you with what you are achieving in life?

0 _________________________[__]______________________________ 10

16) How satisfied are you with your personal relationships?

0 ______________________________[__]____________________________________ 10

17) How satisfied are you with how safe you feel?

0 ______________________________[__]____________________________________ 10

18) How satisfied are you with feeling part of your community?

0 ______________________________[__]____________________________________ 10

19) How satisfied are you with your future security?

0 ______________________________[__]____________________________________ 10

20) How satisfied are you with your spirituality and religion?

0 ______________________________[__]____________________________________ 10

About your health and wellbeing

21) In general would you say your health is:

( ) 1 - excellent

( ) 2 - very good

( ) 3 - good

( ) 4 - fair

( ) 5 - poor

22) For how long (if at all) has your health limited you in each of the following activities?

	**Limited for more than 3 months**	**Limited for less than 3 months**	**Not limited at all**
The kinds or amounts of vigorous activities you can do, like lifting heavy objects, running or participating in strenuous sports	( )	( )	( )
The kinds or amounts of moderate activities you can do, like moving a table, carrying groceries, or bowling	( )	( )	( )
Walking uphill or climbing a few flights of stairs	( )	( )	( )
Bending, lifting, or stooping	( )	( )	( )

Walking one block	( )	( )	( )
Eating, dressing, bathing, or using the toilet	( )	( )	( )

23) How much bodily pain have you had during the past 4 weeks:

( ) 1 - None

( ) 2 - Very mild

( ) 3 - Mild

( ) 4 - Moderate

( ) 5 - Severe

( ) 6 - Very Severe

24) Does your health keep you from working at a job, doing work around the house, or going to school?

( ) 1 - YES, for more than 3 months

( ) 2 - YES, for 3 months or less

( ) 3 - NO

25) Have you been unable to do certain kinds or amounts of work, housework, or schoolwork because of your health?

( ) 1 - YES, for more than 3 months

( ) 2 - YES, for 3 months or less

( ) 3 - NO

26) For each of the following questions, please mark the circle for the one answer that comes closest to the way you have been feeling during the past month.

	**All of the time**	**Most of the time**	**A good bit of the time**	**Some of the time**	**A little of the time**	**None of the time**
How much of the time, during the past month, has your health limited your social activities (like visiting with friends or close relatives)?	( )	( )	( )	( )	( )	( )
How much of the time, during the past month, have you been a very nervous person?	( )	( )	( )	( )	( )	( )
During the past month, how much of the time have you felt calm and peaceful?	( )	( )	( )	( )	( )	( )
How much of the time, during the past month, have you felt downhearted and blue?	( )	( )	( )	( )	( )	( )
During the past month, how much of the time have you been a happy person?	( )	( )	( )	( )	( )	( )

How often, during the past month, have you felt so down in the dumps that nothing could cheer you up?	( )	( )	( )	( )	( )	( )

27) Please select the answer that describes whether the following statements is true or false for you.

	**Definitely true**	**Mostly true**	**Not sure**	**Mostly false**	**Definitely false**
I am somewhat ill	( )	( )	( )	( )	( )
I am as healthy as anybody I know	( )	( )	( )	( )	( )
My health is excellent	( )	( )	( )	( )	( )
I have been feeling bad lately	( )	( )	( )	( )	( )

About Your Health and Wellbeing

28) In the last 3 years, have you been diagnosed or treated for: (Mark all that apply)

[ ] Type 1 diabetes

[ ] Non-insulin dependent Type 2 diabetes

[ ] Insulin dependent Type 2 diabetes

[ ] Cancer – benign

[ ] Cancer – malignant

[ ] Heart disease

[ ] Hypertension (high blood pressure)

[ ] Dyslipidaemia (high cholesterol and/or triglycerides

[ ] Osteoarthritis

[ ] Other musculoskeletal disorder

[ ] Asthma

[ ] Bronchitis

[ ] Other respiratory disorder

[ ] Endometriosis

[ ] Polycystic ovarian syndrome

[ ] Other female reproductive disorder

[ ] Benign prostatic hyperplasia

[ ] Other male reproductive disorder

[ ] Irritable bowel syndrome

[ ] Inflammatory bowel disease

[ ] Celiac disease

[ ] Gastro-oesophageal reflux disease (GERD)

[ ] Chronic constipation

[ ] Other gastrointestinal/digestive disorder

[ ] Mood disorder (e.g. depression)

[ ] Anxiety disorder

[ ] Sleep disorder

[ ] Substance use disorder

[ ] Schizophrenia or other psychotic disorder

[ ] Other mental health disorder

[ ] Other health condition - please specify: ____

( ) None of the above

29) In the last year, have you visited a doctor concerned about your health?

( ) Yes

( ) No

( ) Unsure

30) What was the outcome of your visit with your medical doctor? (select all that apply)

[ ] I was provided an adequate explanation of my health complaint

[ ] I was provide a formal diagnosis of my health condition

[ ] I was prescribed an acceptable treatment plan to manage my health complaint

[ ] I am still bothered by the same health concern

[ ] I am bothered by a new health concern

[ ] Other - please specify: ____*

About your use of health services

31) What was the frequency, reason and cost for visiting the following health professionals in the previous 12 months? Please select the responses that best apply to you.

	**Number of visits**					**Reason for visit (select all that apply)**				**Estimated out-of-pocket expenses per visit ($)** ***Consultation fees only***
	**None**	**1-2**	**3-4**	**5-6**	**More than 6**	**For an acute illness/condition, one that lasted less than one month**	**To treat a long-term health condition (one that lasted more than one month) or its symptoms)**	**To improve wellbeing**	**Other (please specify)**	
A family doctor or another General practitioner (GP)	( )	( )	( )	( )	( )	[ ]	[ ]	[ ]	[ ]	___
A specialist doctor	( )	( )	( )	( )	( )	[ ]	[ ]	[ ]	[ ]	___
A hospital doctor (in outpatients or casualty)	( )	( )	( )	( )	( )	[ ]	[ ]	[ ]	[ ]	___

A pharmacist	( )	( )	( )	( )	( )	[ ]	[ ]	[ ]	[ ]	___
A counsellor of other mental health worker	( )	( )	( )	( )	( )	[ ]	[ ]	[ ]	[ ]	___
A chiropractor	( )	( )	( )	( )	( )	[ ]	[ ]	[ ]	[ ]	___
An osteopath	( )	( )	( )	( )	( )	[ ]	[ ]	[ ]	[ ]	___
A massage therapist	( )	( )	( )	( )	( )	[ ]	[ ]	[ ]	[ ]	___
An acupuncturist	( )	( )	( )	( )	( )	[ ]	[ ]	[ ]	[ ]	___
A naturopath	( )	( )	( )	( )	( )	[ ]	[ ]	[ ]	[ ]	___
A Western herbalist	( )	( )	( )	( )	( )	[ ]	[ ]	[ ]	[ ]	___
A traditional Chinese medicine practitioner	( )	( )	( )	( )	( )	[ ]	[ ]	[ ]	[ ]	___
A homeopath	( )	( )	( )	( )	( )	[ ]	[ ]	[ ]	[ ]	___
An aromatherapist	( )	( )	( )	( )	( )	[ ]	[ ]	[ ]	[ ]	___
A community nurse	( )	( )	( )	( )	( )	[ ]	[ ]	[ ]	[ ]	___
A physiotherapist	( )	( )	( )	( )	( )	[ ]	[ ]	[ ]	[ ]	___
A yoga teacher	( )	( )	( )	( )	( )	[ ]	[ ]	[ ]	[ ]	___
Other (please specify)__________	( )	( )	( )	( )	( )	[ ]	[ ]	[ ]	[ ]	___

32) If you used any of the following treatments in the previous 12 months, who prescribed them to you and how much did you spend on them? Please select the responses that best apply to you.

	**Source of prescription**							**Estimated out-of-pocket expenses per visit ($)**
	**Did not use**	**A family doctor or General practitioner (GP)**	**A specialist doctor**	**A pharmacist**	**A pharmacy or health food store assistant**	**An alternative health practitioner**	**Self-prescribed**	***Write amount below – enter 0 if none spent***
Prescription-only pharmaceuticals	[ ]	[ ]	[ ]	[ ]	[ ]	[ ]	[ ]	___
Over-the-counter pharmaceuticals	[ ]	[ ]	[ ]	[ ]	[ ]	[ ]	[ ]	___
Western or Chinese herbal medicines	[ ]	[ ]	[ ]	[ ]	[ ]	[ ]	[ ]	___
Vitamin/mineral supplements	[ ]	[ ]	[ ]	[ ]	[ ]	[ ]	[ ]	___
Yoga, Tai Chi or Qigong	[ ]	[ ]	[ ]	[ ]	[ ]	[ ]	[ ]	___
Aromatherapy oils	[ ]	[ ]	[ ]	[ ]	[ ]	[ ]	[ ]	___

Homeopathy	[ ]	[ ]	[ ]	[ ]	[ ]	[ ]	[ ]	___
Flower essences	[ ]	[ ]	[ ]	[ ]	[ ]	[ ]	[ ]	___
Relaxation techniques/meditation	[ ]	[ ]	[ ]	[ ]	[ ]	[ ]	[ ]	___
Other (please specify) ________________	[ ]	[ ]	[ ]	[ ]	[ ]	[ ]	[ ]	___

33) Do you take prescription medicine daily?

( ) Yes

( ) No

34) What is the medicine taken to treat?

____

____

____

____

Knowledge about alternative medicine

35) Below is a list of statements about *herbal products*. Please indicate the degree to which you agree or disagree with each statement.

	**Strongly agree**	**Agree**	**Neutral**	**Disagree**	**Strongly Disagree**
The federal government sets standards for the quality of herbal products	( )	( )	( )	( )	( )
Herbal products come in a variety of forms, for example, liquid, lotion, pills	( )	( )	( )	( )	( )
Herbal products are readily available in a variety of stores	( )	( )	( )	( )	( )
There is enough information on the herbal product label to make a well-informed choice about using it	( )	( )	( )	( )	( )
Herbal products sold in Australia may be made in foreign countries	( )	( )	( )	( )	( )
It does not matter how often an herbal product is taken	( )	( )	( )	( )	( )
There is no need to inform a health care provider about taking herbal products	( )	( )	( )	( )	( )
Herbal products do not have side effects	( )	( )	( )	( )	( )

The law requires that the label on the herbal product contain information about what the product is supposed to do	( )	( )	( )	( )	( )
If a famous person recommends a herbal product, it must work	( )	( )	( )	( )	( )
The AUST R and AUST L numbers mean that there has been quality control in the manufacture of the herbal product	( )	( )	( )	( )	( )
The words “organic” and “natural” mean the same thing	( )	( )	( )	( )	( )
If a herbal product is helpful for a friend or family member it will help me	( )	( )	( )	( )	( )
Herbal products can prevent most health problems	( )	( )	( )	( )	( )
The way herbal products work in the body is often not known	( )	( )	( )	( )	( )
It is important to know the correct amount of a herbal product to be used	( )	( )	( )	( )	( )
There is plenty of good information about the quality of herbal products	( )	( )	( )	( )	( )
Many herbal products can be purchased over the Internet	( )	( )	( )	( )	( )
There are few research studies showing that herbal products work	( )	( )	( )	( )	( )

There is little research about the safety of herbal products	( )	( )	( )	( )	( )
It is important to keep track of what happens after starting to take a herbal product	( )	( )	( )	( )	( )

36) Below is a list of statements about *vitamin supplements*. Please indicate the degree to which you agree or disagree with each statement.

	Strongly agree	Agree	Neutral	Disagree	Strongly Disagree
The federal government sets the standards for the quality of vitamin supplements	( )	( )	( )	( )	( )
Vitamin supplements come in a variety of forms, for example, liquid, powder, pills	( )	( )	( )	( )	( )
Vitamin supplements are readily available in a variety of stores	( )	( )	( )	( )	( )
There is enough information on the vitamin supplement label to make a well-informed choice about using it	( )	( )	( )	( )	( )
Vitamin supplement sold in Australia may be made in foreign countries	( )	( )	( )	( )	( )

It does not matter how often a vitamin supplement is taken	( )	( )	( )	( )	( )
There is no need to inform a health care provider about taking vitamin supplements	( )	( )	( )	( )	( )
Vitamin supplements do not have side effects	( )	( )	( )	( )	( )
The law requires that the label on the vitamin supplement contain information about what the product is supposed to do	( )	( )	( )	( )	( )
If a famous person recommends a vitamin supplement, it must work	( )	( )	( )	( )	( )
The AUST R and AUST L numbers mean that there has been quality control in the manufacture of the herbal product	( )	( )	( )	( )	( )
If a vitamin supplement is helpful for a friend or family member it will help me	( )	( )	( )	( )	( )
Vitamin supplements can prevent most health problems	( )	( )	( )	( )	( )
The way vitamin supplements work in the body is often not known	( )	( )	( )	( )	( )
It is important to know the correct amount of a vitamin supplement to be used	( )	( )	( )	( )	( )

There is plenty of good information about the quality of vitamin supplements	( )	( )	( )	( )	( )
Many vitamin supplements can be purchased over the internet	( )	( )	( )	( )	( )
There are few research studies showing that vitamin supplements work	( )	( )	( )	( )	( )
There is little research about the safety of vitamin supplements	( )	( )	( )	( )	( )
It is important to keep track of what happens after starting to take a vitamin supplement	( )	( )	( )	( )	( )

37) Below is a list of statements about *alternative medicine practitioners*. Please indicate the degree to which you agree or disagree with each statement.

	**Strongly agree**	**Agree**	**Neutral**	**Disagree**	**Strongly Disagree**
The federal government sets the standards for the practice requirements of all alternative medicine practitioners	( )	( )	( )	( )	( )

There is enough information available about alternative medicine practitioners to make a well-informed choice about consulting with them	( )	( )	( )	( )	( )
Alternative medicine practitioners practicing in Australia may be trained in foreign countries	( )	( )	( )	( )	( )
Treatments received from an alternative medicine practitioner do not have risks of side effects	( )	( )	( )	( )	( )
The law requires that alternative medicine practitioners provide accurate information about what their treatments are supposed to do	( )	( )	( )	( )	( )
If a particular type of alternative medicine practitioner is helpful for a friend or family member they will help me	( )	( )	( )	( )	( )
Alternative medicine practitioners can treat most health problems	( )	( )	( )	( )	( )
There is no need to inform an alternative medicine practitioner about the medicines I take	( )	( )	( )	( )	( )
There are channels I can use to report any concerns I have about the practices of an alternative medicine practitioner	( )	( )	( )	( )	( )
There are few research studies showing that alternative medicine treatments work	( )	( )	( )	( )	( )
There is little research about the safety of alternative medicine practitioners	( )	( )	( )	( )	( )

I don’t understand the differences between the various types of alternative practitioners	( )	( )	( )	( )	( )

Section 5:

Accessing and sharing information about alternative medicine

38) The following questions relate to the disclosure of information about your use of health services to health professionals providing your health care.

Please select the response that best reflects your experience with the following health professionals in the previous 12 months

Complementary and alternative medicines are treatments not normally considered part of mainstream healthcare, such as herbal medicine, nutritional supplements and homeopathy.

	I told them about ALL complementary and alternative medicines I was using	I only told them about SOME of my complementary and alternative medicine use	I DID NOT tell them about my complementary and alternative medicine use	I did not visit this type of health professional
A family doctor or general practitioner (GP)	( )	( )	( )	( )
A specialist doctor	( )	( )	( )	( )
A hospital doctor (in outpatients or casualty)	( )	( )	( )	( )

A pharmacist	( )	( )	( )	( )

39) The following questions relate to your interactions with a medical doctor in the last 12 months. Please indicate your level of agreement with the following statements (1 = Strongly disagree to 5 = Strongly agree)

I did not disclose my complementary and alternative medicine use to my medical doctor because…

[ ] They did not ask me about my alternative medicine use

[ ] I did not think it was important

[ ] I did not think they would understand my choice

[ ] I was worried they would judge me

[ ] Complementary and alternative medicines are safe

[ ] They did not need to know

[ ] There was not enough time in the consultation

[ ] I felt uncomfortable discussing it with them

[ ] I was worried they wouldn’t support my treatment decisions

[ ] I did not think they would know anything about complementary and alternative medicine

[ ] I forgot to mention it

[ ] It is none of their business

[ ] I was worried they would try to discourage my use of complementary and alternative medicine

[ ] They do not approve of my use of complementary and alternative medicine

[ ] I was worried they would respond negatively

[ ] I do not use complementary and alternative medicine regularly enough

[ ] I previously had a negative experience when I disclosed using complementary and alternative medicine

I disclosed my complementary and alternative medicine use to my health practitioner because…

[ ] I wanted them to fully understand my health status

[ ] I was concerned about drug interactions with the complementary and alternative medicine I was using

[ ] I thought they might know something about complementary and alternative medicine

[ ] They asked me about my use of complementary and alternative medicine

[ ] I have a good relationship with them

[ ] I felt comfortable discussing complementary and alternative medicine with them

[ ] I knew they would be willing to discuss my alternative medicine use

[ ] I wanted their approval of my complementary and alternative medicine use

[ ] I knew they would understand about my complementary and alternative medicine use

[ ] They have a good attitude towards complementary and alternative medicine

[ ] They are open-minded

[ ] I thought they could help with my treatment decisions

[ ] They support my use of complementary and alternative medicines

[ ] They understand my treatment goals

[ ] They have my best interests at heart

[ ] I wanted their advice about complementary and alternative medicines

39) The following questions relate to the disclosure of information about your use of conventional health services to alternative medicine practitioners providing you health care.

Please select the response that best reflects your experience with the following health professionals in the previous 12 months

	I told them about ALL conventional medicines I was using	I only told them about SOME of my conventional medicine use	I DID NOT tell them about my conventional medicine use	I did not visit this type of health professional
Massage therapist	( )	( )	( )	( )
Acupuncturist	( )	( )	( )	( )
Naturopath	( )	( )	( )	( )
Western herbalist	( )	( )	( )	( )
Traditional Chinese medicine practitioner	( )	( )	( )	( )
Homeopath	( )	( )	( )	( )
Chiropractor	( )	( )	( )	( )

Conventional medicines are treatments that are considered part of mainstream healthcare, such as pharmaceutical drugs.

41) The following questions relate to your interactions with complementary and alternative health practitioners (e.g. naturopath, herbalist, homeopath) in the last 12 months. Please indicate your level of agreement with the following statements (1 = Strongly disagree to 5 = Strongly agree)

I did not disclose my conventional medicine use to my complementary and alternative health practitioner because…

[ ] They did not ask me about my conventional medicine use

[ ] I did not think it was important

[ ] I did not think they would understand my choice

[ ] I was worried they would judge me

[ ] They did not need to know

[ ] There was not enough time in the consultation

[ ] I felt uncomfortable discussing it with them

[ ] I was worried they wouldn’t support my treatment decisions

[ ] I did not think they would know anything about conventional medicine

[ ] I forgot to mention it

[ ] It is none of their business

[ ] I was worried they would try to discourage my use of conventional medicine

[ ] They do not approve of my use of conventional medicine

[ ] I was worried they would respond negatively

[ ] I do not use conventional medicines regularly enough

[ ] I previously had a negative experience when I disclosed using conventional medicine

I disclosed my conventional medicine use to my complementary and alternative health practitioner because…

[ ] I wanted them to fully understand my health status

[ ] I was concerned about drug interactions with the conventional medicine I was using

[ ] I thought they might know something about conventional medicines

[ ] They asked me about my use of conventional medicine

[ ] I have a good relationship with them

[ ] I felt comfortable discussing conventional medicine with them

[ ] I knew they would be willing to discuss my conventional medicine use

[ ] I wanted their approval of my conventional medicine use

[ ] I knew they would understand about my conventional medicine use

[ ] They have a good attitude towards conventional medicine

[ ] They are open-minded

[ ] I thought they could help with my treatment decisions

[ ] They support my use of conventional medicines

[ ] They understand my treatment goals

[ ] They have my best interests at heart

[ ] I was concerned about side-effects of conventional medicines

[ ] I wanted their advice about conventional medicines

Thank You!

Thank you for taking our survey. Your response is very important to us.
